# Beneficial effect of adjuvant traditional Chinese medicine therapy on body constitution symptoms and quality of life among breast cancer patients

**DOI:** 10.3389/fonc.2026.1734421

**Published:** 2026-04-20

**Authors:** Chien-Wei Tseng, Ya-Ting Hsu, Jing-Shiang Hwang, Yao-Jen Chang, Hsien-Chang Wu

**Affiliations:** 1 Department of Chinese Medicine, Taipei Tzu Chi Hospital, The Buddhist Tzu Chi Medical Foundation, New Taipei City, Taiwan; 2 School of Post-baccalaureate Chinese Medicine, Tzu Chi University, Hualien, Taiwan; 3 Institute of Statistical Science, Academia Sinica, Taipei, Taiwan; 4 Department of Surgery, Taipei Tzu Chi Hospital, The Buddhist Tzu Chi Medical Foundation, New Taipei City, Taiwan; 5 School of Medicine, Tzu Chi University, Hualien, Taiwan

**Keywords:** body constitution, breast cancer, quality of life, real-world study, traditional Chinese medicine

## Abstract

**Background:**

Breast cancer therapies effectively control tumors but frequently impose substantial symptom burden and impair quality of life (QOL). In integrative oncology practice, traditional Chinese medicine (TCM) is commonly used as adjunctive supportive care. We evaluated associations between adjunctive scientific Chinese herbal medicine (CHM), constitution-related symptoms, and QOL in a real-world breast cancer cohort.

**Methods:**

In this prospective observational study, 83 patients receiving Western medicine (WM) were followed for 3–6 months and categorized as WM alone or WM plus adjunctive CHM. Baseline imbalance was addressed using inverse probability of treatment weighting (IPTW), and repeated measures were analyzed using generalized estimating equation (GEE) models to estimate associations between CHM exposure and improvements in BCQ and WHOQOL-BREF outcomes.

**Results:**

After IPTW adjustment, adjunctive CHM use was associated with higher odds of improvement in BCQ symptoms including fatigue (OR 1.92, 95% CI 1.18–3.12), dry eyes (OR 2.15, 95% CI 1.27–3.65), and hot flushes (OR 1.87, 95% CI 1.10–3.19), compared with WM alone. Associations were also observed in WHOQOL-BREF, including reduced interference from physical pain (OR 2.08, 95% CI 1.24–3.50) and improved perception of physical environment (OR 1.69, 95% CI 1.03–2.76). These symptom patterns are consistent with traditional Chinese medicine descriptions commonly characterized as qi–yin deficiency with stasis-stagnation.

**Conclusion:**

In this real-world integrative oncology setting, adjunctive scientific CHM use was associated with improvements in constitution-related symptoms and selected QOL domains. These findings suggest a potential supportive role for CHM in breast cancer care; however, causal inference cannot be established.

## Introduction

Breast cancer is the most frequently diagnosed malignancy among women worldwide. In 2022, approximately 2.3 million new cases were reported, representing 11.6% of all newly diagnosed cancers globally (1, 2). Despite advances in screening and treatment strategies, breast cancer remains a leading cause of cancer-related mortality, continuing to pose a significant public health challenge. This has driven the search for more effective prevention, treatment, and personalized therapy approaches (3).

Western medicine (WM) remains the primary treatment modality for breast cancer, including chemotherapy, radiotherapy, targeted therapy, and immunotherapy. While chemotherapy agents like doxorubicin, paclitaxel, and cyclophosphamide inhibit tumor cell division, they also induce cytotoxic effects on normal rapidly dividing cells, leading to side effects such as nausea, vomiting, hair loss, cognitive impairment, and bone marrow suppression (4, 5). Radiotherapy, though effective for localized lesions, can cause side effects such as skin erythema and fatigue (6). Targeted therapy, such as trastuzumab for HER2-positive breast cancer, improves drug specificity but can result in severe side effects like congestive heart failure and diarrhea (7). While effectively stimulating immune responses against tumors, immunotherapy may also cause systemic inflammatory reactions and autoimmune responses (8). Though essential for tumor inhibition, these WM therapies often impact patients’ quality of life (QOL), necessitating research into mitigating these adverse effects (9, 10).

Adjuvant traditional Chinese medicine (TCM) has garnered attention for its potential to reduce the side effects of WM therapies. TCM theory suggests that WM therapies disrupt the body’s constitutional balance, causing symptoms such as fatigue, insomnia, and appetite loss. Systematic reviews and randomized controlled trials (RCTs) have reported potential benefits of integrating TCM with conventional therapies, including associations with improved survival, reducing chemotherapy-related adverse effects, and enhanced QOL (11–15). However, these studies are often conducted in controlled environments with strict eligibility criteria, which may limit generalizability to routine clinical practice (16, 17). Moreover, most investigations focus on survival or recurrence rather than constitution-related symptom patterns from a TCM perspective. The lack of real-world evidence has hindered the development of personalized TCM strategies based on syndrome differentiation and treatment strategies.

This study aimed to explore whether adjuvant CHM use was associated with improvements in constitution-related symptoms and QOL among breast cancer patients receiving WM, using a prospective real-world observational framework. The findings may provide new clinical insights and evidence to inform comprehensive treatment strategies for breast cancer.

## Subjects and methods

This study employed a prospective observational cohort design without randomization or blinding. Breast cancer patients who consented to participate and signed the consent form were invited. Clinical staging and WM treatment information were obtained through retrospective medical record review, whereas patient-reported outcomes were collected prospectively using standardized questionnaires. Treatments and patient characteristics were considered independent variables, while QOL and TCM body constitution scores were the dependent variables.

Patients were recruited by convenience sampling, including those receiving WM therapy alone and those receiving both WM and adjuvant CHM as part of routine clinical care. The study was approved by the Institutional Review Board of Taipei Tzu Chi Hospital (Protocol No.: 05-X21-083). Researchers provided oral and written explanations to eligible participants in TCM and WM clinics; after obtaining written informed consent, participants were included for assessment and follow-up. Between January 1, 2018, and June 30, 2019, a total of 157 breast cancer patients were initially recruited from the Breast Surgery, Oncology, and TCM departments. Six patients were excluded due to incomplete data in key variables (WHOQOL-BREF or BCQ), leaving 151 participants for the final analysis. Of these, 63 received WM-only therapy and 88 received WM plus adjuvant TCM. Data were collected at baseline, 3 months, and 6 months using the Taiwan versions of the WHOQOL-BREF and the Body Constitution Questionnaire (BCQ) (**Supplementary Files S1**, **S2**).

### Eligibility criteria

- Inclusion: (1) age ≥18 years; (2) diagnosed with breast cancer and receiving WM treatment at Taipei Tzu Chi Hospital; (3) completed WHOQOL-BREF and BCQ assessments at baseline, 3, and 6 months; (4) for the adjuvant TCM group, received ≥28 days of CHM therapy during WM treatment.
- Exclusion: (1) refusal or inability to provide informed consent; (2) patients undergoing diagnostic evaluation only or seeking second opinion consultation; (3) incomplete data preventing outcome assessment.

### Definition of treatments

WM therapy was defined as conventional treatment for breast cancer (chemotherapy, targeted therapy, radiotherapy, or hormone therapy). Adjuvant TCM exposure was defined as receipt of ≥28 cumulative days of scientific Chinese herbal medicine (CHM) during the course of WM treatment; acupuncture was not included. CHM prescriptions were typically administered three times daily after meals, according to routine clinical practice. Acupuncture was not included in this definition. If patients continued hormone therapy after chemo-radiotherapy, the definition of adjuvant CHM therapy remained the same.

### Timing of treatment exposure and assessments

In our clinical setting, patients were diagnosed with breast cancer by surgical oncologists and referred to the TCM outpatient clinic or consultation service for supportive care during the course of Western medical treatment. The first questionnaire assessment (baseline) was conducted at the time of study enrollment. In most cases, adjunctive TCM treatment was initiated after the baseline assessment. Therefore, the baseline evaluation generally preceded or closely coincided with the initiation of TCM therapy. Subsequent questionnaire assessments were conducted during follow-up visits after initiation of TCM treatment. This temporal ordering ensured that baseline symptom measurements were recorded before or at the early stage of TCM exposure.

### Outcome measures

- Primary outcome: Improvement in constitution-related symptoms measured by the BCQ, a 44-item instrument based on TCM theory, categorizing individuals into Yin-deficiency, Yang-deficiency, and Phlegm-stasis constitution types. Each item is rated from 1 to 5, with total scores ranging from 0 to 220. The BCQ has been shown to have robust content validity (CVI: 0.73–1.00), internal consistency (Cronbach’s α: 0.62–0.88), and test-retest reliability (ICC > 0.7) in previous Taiwanese studies (20, 21).
- Secondary outcomes: Quality of life assessed by the WHOQOL-BREF (Taiwan version), a 28-item questionnaire assessing four domains of quality of life, including physical health, psychological well-being, social relationships, and environment. Items are rated on a 5-point Likert scale, with domain scores ranging from 4 to 20, where higher scores indicate better perceived QOL. The instrument has been shown to have strong reliability and validity in Taiwanese populations (18, 19).

Improvement was defined as a higher follow-up score compared with baseline for WHOQOL-BREF and a lower follow-up score for BCQ, consistent with instrument scoring properties. The English versions of the BCQ and WHOQOL-BREF questionnaires used in this study are provided in the **Supplementary File S1**.

### Reporting guideline

This study adhered to the STROBE statement for observational studies.

### Statistical analysis

The categorical variables are presented as frequencies and percentages. The distribution of participants’ baseline characteristics across the two study groups (the WM and WM+TCM groups) may exhibit imbalance. To quantify this, we calculated the standardized mean difference (SMD) for each characteristic. Analysis of the original sample reveals that most baseline characteristics have SMD values exceeding 0.1, indicating a significant imbalance (**Table 1**). To address this, we used the Inverse Probability of Treatment Weighting (IPTW) method to balance the comparative groups. This approach involves assigning a weight to each participant, which is the inverse of their probability or propensity score of being allocated to their original group. These scores are estimated using a logistic regression model based on their baseline characteristics. We then reassessed the weighted samples using SMD to confirm the absence of significant differences in baseline characteristics between the two adjusted groups (**Table 1**).

**Table 1:** Baseline characteristics of the western medicine and adjuvant traditional chinese medicine groups before and after inverse probability of treatment weighting.

Characteristics	Original samples			IPTW samples		
	WM	WM+TCM	SMD	WM	WM+TCM	SMD
	N=63(%)	N=88 (%)				
Age:						
30-49	20.0 (31.7)	24.0 (27.3)	0.139	44.4 (29.2)	41.8 (27.6)	0.047
50-59	21.0 (33.3)	35.0 (39.8)		53.6 (35.3)	52.9 (34.9)	
≥60	22.0 (34.9)	29.0 (33.0)		53.7 (35.4)	56.9 (37.5)	
Occupation:						
Employed	26.0 (41.3)	29.0 (33.0)	0.194	46.8 (30.8)	49.1 (32.4)	0.064
Homemaker	18.0 (28.6)	32.0 (36.4)		59.9 (39.5)	55.1 (36.4)	
Others	19.0 (30.2)	27.0 (30.7)		45.0 (29.7)	47.3 (31.2)	
Marital status:						
Married	41.0 (65.1)	65.0 (73.9)	0.332	111.2 (73.3)	106.9 (70.6)	0.062
Unmarried	8.0 (12.7)	14.0 (15.9)		17.4 (11.5)	19.5 (12.9)	
Others	14.0 (22.2)	9.0 (10.2)		23.1 (15.2)	25.1 (16.6)	
Religion:						
Buddhism	37.0 (58.7)	68.0 (77.3)	0.484	104.3 (68.8)	103.6 (68.4)	0.009
Catholicism/Christianity	6.0 (9.5)	1.0 (1.1)		7.1 (4.7)	7.1 (4.7)	
Others	20.0 (31.7)	19.0 (21.6)		40.3 (26.5)	40.8 (26.9)	
Education:						
illiteracy/elementary/middle	18.0 (28.6)	26.0 (29.5)	0.080	52.6 (34.7)	46.9 (31.0)	0.081
High school	19.0 (30.2)	29.0 (33.0)		44.0 (29.0)	45.4 (30.0)	
College or above	26.0 (41.3)	33.0 (37.5)		55.0 (36.3)	59.2 (39.1)	
BMI:						
<18.5	3.0 (4.8)	3.0 (3.4)	0.229	5.2 (3.5)	5.7 (3.8)	0.042
18.5≤BMI<24	21.0 (33.3)	39.0 (44.3)		60.8 (40.1)	57.7 (38.1)	
BMI≥24	39.0 (61.9)	46.0 (52.3)		85.7 (56.5)	88.1 (58.1)	
Cancer stage:						
Carcinoma In Situ	5.0 (7.9)	5.0 (5.7)	0.098	9.6 (6.3)	10.2 (6.7)	0.016
I, II	43.0 (68.3)	63.0 (71.6)		106.4 (70.1)	106.3 (70.2)	
III, IV	15.0 (23.8)	20.0 (22.7)		35.7 (23.5)	35.0 (23.1)	
Cancer treatment:						
Chemotherapy	31.0 (49.2)	45.0 (51.1)	0.039	73.6 (48.5)	77.4 (51.1)	0.052
Others	32.0 (50.8)	43.0 (48.9)		78.1 (51.5)	74.1 (48.9)	
Duration: (years)						
≤0.5	42.0 (66.7)	46.0 (52.3)	0.329	86.6 (57.1)	88.8 (58.6)	0.064
>0.5 and ≤1	6.0 (9.5)	8.0 (9.1)		12.4 (8.2)	14.2 (9.4)	
>1	15.0 (23.8)	34.0 (38.6)		52.7 (34.7)	48.6 (32.0)	

WM, Western Medicine; WM+TCM, Western Medicine combined with adjuvant traditional Chinese Medicine; IPTW, inverse probability of treatment weighting; SMD: standardized mean difference; BMI, body mass index. Data are present as n (%). A SMD ≤ 0.1 indicates a negligible difference between the two study groups.

We defined binary response variables to assess whether the measured scores of BCQ/WHOQOL at the second and third evaluations show improvement compared to the first evaluation. We denote Z_ij_=1 if the score at the j-th evaluation for the i-th participant has improved, and 0 otherwise, for j=2, 3. For the two repeated measurements of each participant, we proposed a generalized estimating equations (GEE) model to fit the weighted samples. The GEE model is specified as follows:


$$\begin{array}{c} \text{logit }[\text{Pr }{(\text{Z}}_{\text{ij}}\;=\;1)]\;=\;\alpha_{0}\;+\;\\ \sum_{\text{k}}\alpha_{\text{k}}\;\times \;{\text{X}}_{\text{ik}}\;+\;\sum_{\text{h}=1, 3}{\text{β}}_{1\text{h}}\;\times \text{ I }{(}_{\text{Si}}\;=\text{ h})\;+\;{\text{β}}_{2}\;\times \text{ I }\left({\text{T}}_{\text{i}}\;=1\right)\\ \;+\;\sum_{\text{h}=1,\;3}{\text{γ}}_{1\text{h}}\;\times \;\left[{\text{G}}_{\text{i}}:\text{I}\left({\text{S}}_{\text{i}}=\text{h}\right)\right]\;+\;{\text{γ}}_{2}\;\\ \times \;\left[{\text{G}}_{\text{i}}:\text{I}\left({\text{T}}_{\text{i}}=1\right)\right]\\ +\;{\text{γ}}_{3}\;\times \;{\text{G}}_{\text{i}} \end{array}$$

where Z_i2_ and Z_i3_ are correlated. The independent variable X_ik_ refers to baseline characteristics. The term S_i_=1, 2, 3 indicates cancer stage of 0, I-II and III-IV, respectively. T_i_ denotes whether the participant received chemotherapy, and G_i_ indicates TCM intervention. The I (•) is an indicator function. The term [U:V] represents interaction of variables U and V. We utilized the R package glmtoolbox to estimate the parameters in the GEE model.

Note that we assume that the reference category in the GEE model are the patients with stage I-II and no chemotherapy. Using the estimated parameters, the odds ratio (OR) of improvement of TCM versus no TCM intervention for all patients was calculated as.


$$\begin{array}{c} \text{OR}=\exp({\hat{\gamma }}_{11}\times (\text{proportion}\;\text{of stage }0)+{\hat{\gamma }}_{13}\\ \times (\text{proportion of stage III-IV})+{\hat{\gamma }}_{2}\\ \times (\text{proportion of chemo})+{\hat{\gamma }}_{3}). \end{array}$$

The odds ratio of improvement of TCM intervention among patients with stage I-II and receiving chemotherapy (vs. no chemotherapy) was calculated as $\text{OR}=\exp({\hat{\gamma }}_{2}+{\hat{\gamma }}_{3})$. For stage-stratified analysis, in the absence of chemotherapy, the odds ratios of improvement for patients with stage 0, stage I-II and stage III-IV are calculated by $\exp({\hat{\gamma }}_{11}+{\hat{\gamma }}_{3})$, $\exp({\hat{\gamma }}_{3})$ and $\;\exp({\hat{\gamma }}_{13}+{\hat{\gamma }}_{3})$, respectively.

## Results

A total of 151 breast cancer patients were enrolled, with a mean age of 58.0 years. American Joint Committee on Cancer (AJCC) breast cancer staging, 6.4% had carcinoma *in situ*, 25.5% were stage I, 44.6% stage II, 15.9% stage III, and 7.6% stage IV disease. Among all patients, 45.9% received chemotherapy, 8.9% targeted therapy, 13.4% radiotherapy, 26.1% hormone therapy, and 5.7% were under follow-up observation. Of the 151 patients, 63 received Western medicine (WM) alone and 88 received WM combined with adjunctive TCM (WM+TCM). Before weighting, several baseline characteristics showed imbalance between groups (**Table 1**). After inverse probability of treatment weighting (IPTW), covariate balance was achieved, with all standardized mean differences ≤0.1, indicating adequate comparability between groups. There were also no significant differences between the two weighted groups in terms of cancer staging and Western therapy.

**Figure 1:**
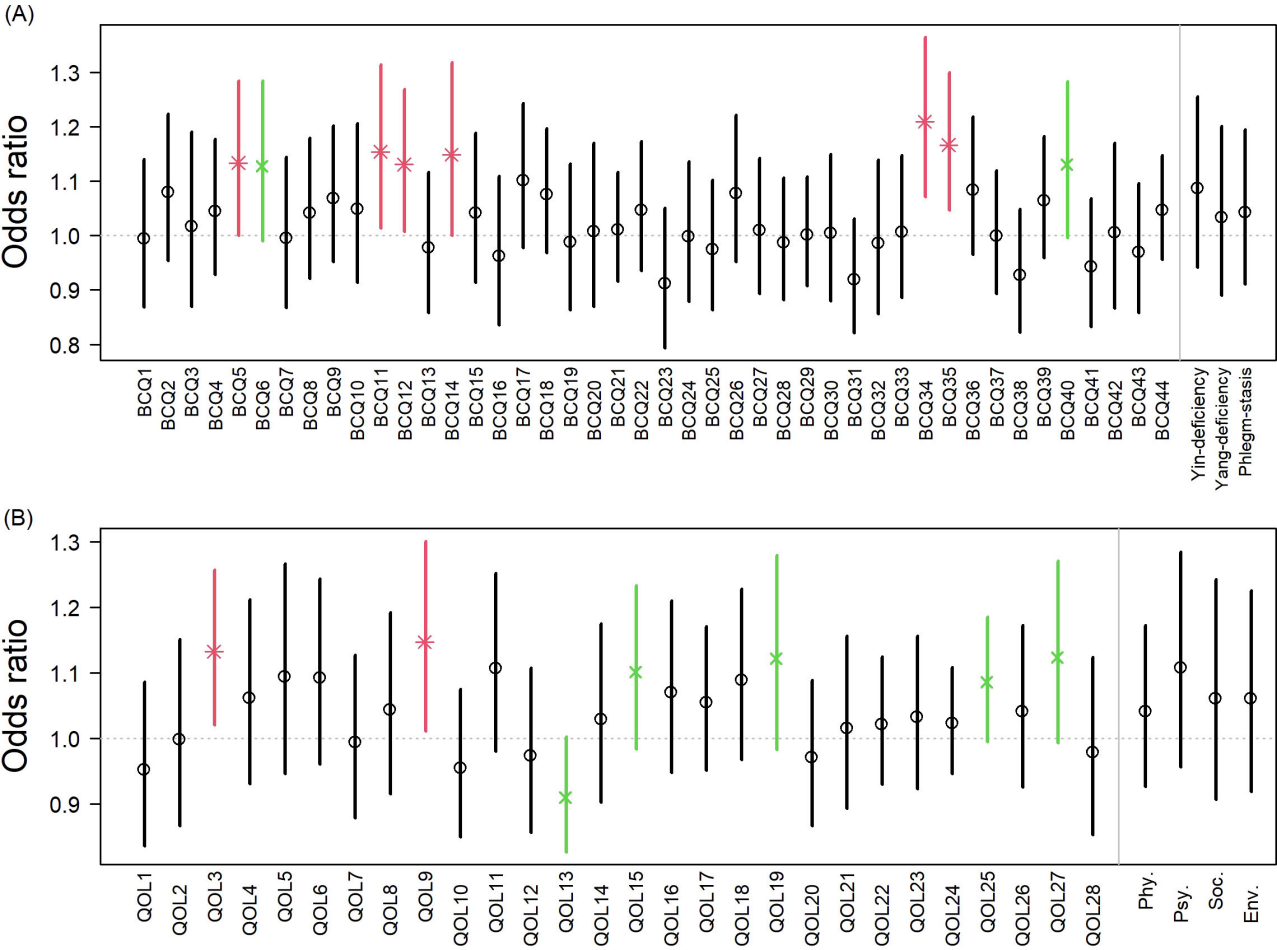
Adjusted odds ratios (ORs) for improvement in BCQ and WHOQOL-BREF items associated with adjuvant CHM use (WM+CHM vs. WM alone). Responses to selected items from the **(A)** Body Constitution Questionnaire (BCQ) and **(B)** WHOQOL-BREF comparing the WM+CHM group with the WM-alone group. Odds ratios (ORs) and 95% confidence intervals (CIs) were estimated using inverse probability of treatment weighting (IPTW)-adjusted generalized estimating equation (GEE) models. Error bars represent 95% CIs. Exact two-sided p-values are reported; * indicates statistical significance (p < 0.05) and × indicates marginal significance (0.05 ≤ p < 0.10). BCQ, Body Constitution Questionnaire; WHOQOL-BREF, World Health Organization Quality of Life-BREF.

**Figure 1** presents adjusted odds ratios (ORs) for improvement associated with adjunctive CHM (WM+CHM vs. WM alone), estimated using IPTW-weighted GEE models with stage I–II and no chemotherapy as the reference. Improvement was defined according to instrument scoring direction (lower BCQ scores and higher WHOQOL-BREF scores at follow-up). Adjunctive CHM use was associated with higher odds of improvement in several BCQ items, including fatigue (BCQ5), dry eyes or blurred vision (BCQ11), dull body pain (BCQ12), sleepiness (BCQ14), thick tongue coating (BCQ34), and hot flushes (BCQ35), with marginal associations observed for thick sticky saliva (BCQ6) and constipation (BCQ40). For WHOQOL-BREF, significant associations were observed for reduced interference from physical pain (QOL3) and improved perception of physical environment (QOL9), with marginal associations in mobility (QOL15), self-satisfaction (QOL19), transport satisfaction (QOL25), and perceived respect from others (QOL27).

**Figure 2:**
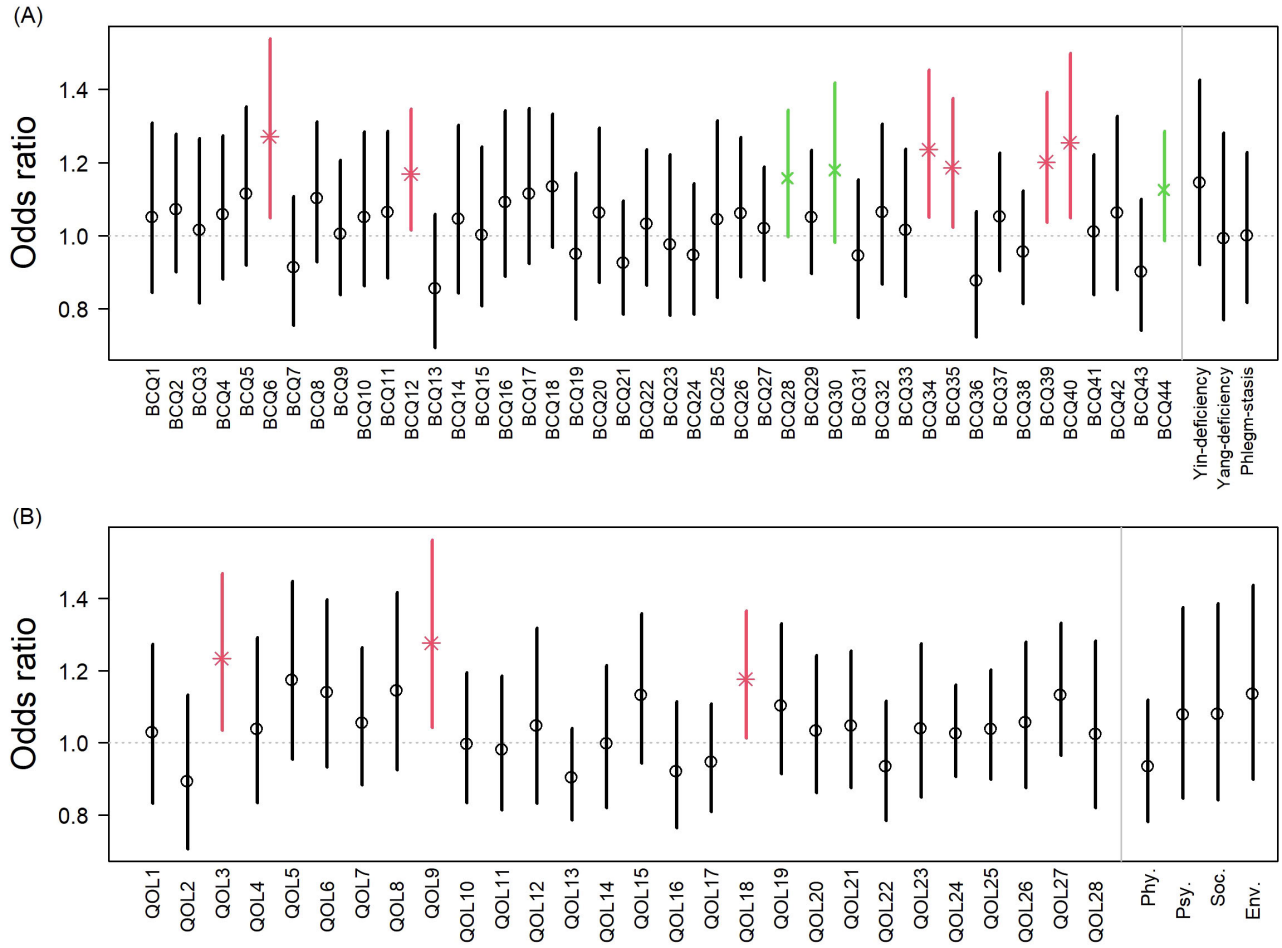
Adjusted odds ratios (ORs) for improvement in BCQ and WHOQOL-BREF items among patients receiving chemotherapy (WM+CHM vs. WM alone). Response to selected items and domains of **(A)** BCQ and **(B)** WHOQOL-BREF among patients undergoing chemotherapy, compared the WM+CHM group with the WM-alone group. Odds ratios (ORs) and 95% confidence intervals (CIs) were estimated using IPTW-adjusted GEE models. Exact two-sided p-values are reported; * indicates statistical significance (p < 0.05) and × indicates marginal significance (0.05 ≤ p < 0.10). Error bars represent 95% confidence intervals. BCQ, Body Constitution Questionnaire; WHOQOL-BREF, World Health Organization Quality of Life-BREF.

To further explore associations among patients receiving chemotherapy, subgroup analyses are shown in **Figure 2**. Within this subgroup of patients with stage I-II cancer, adjunctive CHM use was associated with higher odds of improvement in BCQ items including thick sticky saliva (BCQ6), dry eyes or blurred vision (BCQ11), thick tongue coating (BCQ34), hot flushes (BCQ35), small volume of urine (BCQ39), and constipation (BCQ40). Marginal associations were observed for dyspnea when aggravated by lying flat (BCQ28), dry throat (BCQ30), and diarrhea at dawn (BCQ44). For WHOQOL-BREF, significant associations were observed for reduced interference from physical pain (QOL3), improved perception of physical environment (QOL9), and greater satisfaction with working capacity (QOL18).

Stage-stratified analyses are presented in **Supplementary Figure 1**. Among patients with early-stage disease (stage I–II) and in the absence of chemotherapy, adjunctive CHM use was associated with higher odds of improvement in BCQ items including sleepiness (BCQ14) and preference for warm drinks (BCQ36), with marginal associations observed for hot flushes (BCQ35) and dry mouth or thirst (BCQ26). For WHOQOL-BREF, significant associations were observed for reduced interference from physical pain (QOL3) and greater satisfaction with transportation (QOL25), with marginal associations in coping with daily life during medical treatment (QOL4).

Among patients with advanced-stage disease (stage III–IV) and in the absence of chemotherapy, adjunctive CHM use was associated with higher odds of improvement in BCQ items including dry eyes or blurred vision (BCQ11), sleepiness (BCQ14), and preference for warm drinks (BCQ36), with a marginal association observed for tingling pain in the chest, abdomen, or limbs (BCQ13). For WHOQOL-BREF outcomes, a significant association was observed for improvement in the physical domain score. At the item level, significant associations were observed for satisfaction with sleep (QOL16), ability to perform routine daily activities (QOL17), and satisfaction with social support from friends (QOL22). Marginal associations were observed for health satisfaction (QOL2), acceptance of appearance (QOL11), and satisfaction with transportation (QOL25). Given the reduced sample sizes within stage strata, these findings should be interpreted as exploratory.

Among the patients receiving adjunctive CHM, the number of TCM outpatient visits ranged from 2 to 129. The mean number of visits was 23.2 (SD 18.9), with a median of 22 (IQR 9–33). Analysis of prescribing patterns demonstrated heterogeneous yet structured herbal selection across visits. The most frequently prescribed single herbs were Prunellae Spica (Xia Ku Cao), Curcumae Radix (Yu Jin), Trichosanthis Fructus (Gua Lou Shi), Taraxaci Herba (Pu Gong Ying), and Astragali Radix (Huang Qi). Among compound formulae, Among compound formulae, Sheng Mai Yin (生脈飲) was the most frequently prescribed, followed by Suan Zao Ren Tang (酸棗仁湯), Jia Wei Xiao Yao San (加味逍遙散), Wen Dan Tang (溫膽湯), and Ban Xia Xie Xin Tang (半夏瀉心湯). Detailed rankings, botanical standardization, and therapeutic classifications are provided in **Supplementary Tables S1**, **S2**. Prescribing patterns were consistent with individualized clinical practice.

## Discussion

Breast cancer treatments are effective for tumor control but are frequently accompanied by treatment-related symptoms that adversely affect quality of life. In routine clinical settings, integrative TCM approaches are commonly incorporated as supportive care during WM treatment to address symptom burden. In the present study, adjunctive CHM use was associated with improvements in selected BCQ domains, including fatigue, sleepiness, dry eyes, and hot flushes, as well as WHOQOL-BREF domains related to physical functioning and pain interference. These associations were observed within a real-world cohort and should be interpreted in the context of supportive symptom management rather than causal therapeutic effects.

Within this cohort, the prescribing profile reflected a structured yet individualized supportive-care approach characteristic of integrative oncology practice. Herbal selection varied across visits, consistent with syndrome-based modification during the course of conventional treatment rather than protocol-driven fixed prescriptions. Such flexibility aligns with routine clinical management in which symptom patterns evolve over time during chemotherapy, radiotherapy, or endocrine therapy.

The most frequently prescribed single herbs—Prunellae Spica, Curcumae Radix, Trichosanthis Fructus, Taraxaci Herba, and Astragali Radix—span therapeutic categories including heat-clearing, blood-activating, phlegm-resolving, and qi-tonifying strategies. Among compound formulae, Sheng Mai Yin was most frequently prescribed, followed by Suan Zao Ren Tang, Jia Wei Xiao Yao San, Wen Dan Tang, and Ban Xia Xie Xin Tang. These prescriptions are consistent with symptom-oriented and constitution-based management commonly applied in clinical TCM practice.

Breast cancer could be detected at early stages by hematology test and imaging examination and can be treated with appropriate therapies, including chemotherapy, endocrine and targeted therapy, radiotherapy, immunotherapy and adjuvant hormonal therapy (22, 23). However, these therapies frequently produce treatment-related symptoms and adverse effects (24, 25), which may affect patients’ constitution patterns and overall well-being (26) and ultimately influence quality of life (14).

Previous studies have suggested that TCM used as an adjunct therapy in cancer care may help address treatment-related symptoms and improve patient-reported outcomes (13, 14, 26–29). According to TCM syndrome differentiation, breast cancer patients often present constitution patterns including qi deficiency, blood deficiency, yin deficiency, and yang deficiency (30). Other studies have reported syndrome manifestations such as qi-yin deficiency, liver–kidney deficiency, spleen–kidney deficiency, and deficiency of vital energy and blood during chemotherapy periods (31).

In the present study, adjunctive CHM use was associated with improvements in BCQ symptoms including fatigue, dry eyes, sleepiness, thick tongue coating, and hot flushes, with additional marginal associations in constipation and thick sticky saliva. These symptoms are commonly reported during chemotherapy and radiotherapy, particularly with taxanes and anthracyclines (32, 33). Improvements were also observed in WHOQOL-BREF domains including reduced interference from physical pain and improved perceptions of physical environment and mobility. Collectively, these findings suggest that adjunctive CHM use may play a supportive role in symptom management during active breast cancer treatment.

Subgroup analyses among patients receiving chemotherapy showed similar associations, including improvements in symptoms such as thick saliva, dry eyes, hot flashes, and small urine volume, with additional marginal associations in dyspnea, dry throat, and early-morning diarrhea. Corresponding improvements were observed in WHOQOL-BREF items related to physical pain interference, environmental perception, and work capacity. These findings further suggest potential supportive-care associations of CHM use during systemic cancer therapy.

Stage-stratified analyses suggested associations in both early- and advanced-stage patients, although the domains differed. Early-stage patients showed improvements primarily in pain-related and daily-function domains, whereas advanced-stage patients demonstrated broader associations in sleep, routine activities, and social support domains. Given the modest subgroup sample sizes, these findings should be interpreted as exploratory.

Experimental studies have also explored TCM syndrome differentiation models in cancer research. Animal models have demonstrated associations between TCM-defined syndromes and tumor microenvironment changes (34, 35). Clinical investigations have also reported that breast cancer patients frequently present with qi, blood, yin, or yang deficiency patterns (36), as well as syndrome manifestations such as liver depression with qi stagnation, phlegm and blood stagnation, and spleen deficiency (37). Although mechanistic pathways were not evaluated in the present study, these conceptual frameworks may help contextualize the observed associations within traditional medical theory.

Several limitations should be acknowledged. First, although IPTW adjustment was applied to reduce measured confounding, residual and unmeasured confounding cannot be excluded due to the observational design. Second, although baseline assessments generally preceded or closely coincided with the initiation of TCM therapy, the study cannot fully exclude potential time-related biases, including natural symptom recovery during conventional cancer treatment. Third, CHM exposure was defined by cumulative prescription duration based on medical records, and patient adherence as well as dose–response relationships could not be formally verified. Finally, this was a single-center study with a modest sample size determined by real-world recruitment rather than a formal power calculation, which may limit generalizability.

Accordingly, the findings should be interpreted as associations observed under routine clinical conditions rather than definitive evidence of causal effects. Our study suggests that adjuvant CHM use was associated with improvements in BCQ-related symptoms and selected WHOQOL-BREF domains among breast cancer patients receiving WM therapy. Also, our findings highlight potential supportive-care associations observed with adjuvant CHM use across different cancer stages in breast cancer patients. Furthermore, adjuvant TCM therapies were associated with alleviated symptoms and adverse effects commonly associated with WM therapies, including manifestations related to yin deficiency and phlegm stasis, offering a complementary approach to holistic patient care.

## Conclusion

Among breast cancer patients receiving conventional WM, adjuvant scientific CHM use was associated with improvements in constitution-related symptoms and selected WHOQOL-BREF domains over 3–6 months. While these findings suggest a potential supportive role for integrative care in symptom management and QOL enhancement, causal inference cannot be established. Confirmation in larger, adequately powered prospective or pragmatic trials is warranted.

## Data Availability

The raw data supporting the conclusions of this article will be made available by the authors, without undue reservation.

## Supplementary material

Supplementary Figure 1 Stage-stratified adjusted odds ratios (ORs) for improvement in BCQ and WHOQOL-BREF items associated with adjuvant CHM use. Stage-stratified analyses comparing the WM+CHM group with the WM-alone group. Panels show BCQ outcomes in **(A)** stage I–II and **(C)** stage III–IV, and WHOQOL-BREF outcomes in **(B)** stage I–II and **(D)** stage III–IV. Odds ratios (ORs) and 95% confidence intervals (CIs) were estimated using IPTW-adjusted GEE models. Exact two-sided p-values are reported; * indicates statistical significance (*p* < 0.05) and × indicates marginal significance (0.05 ≤ *p* < 0.10). Error bars represent 95% confidence intervals. BCQ, Body Constitution Questionnaire; WHOQOL-BREF, World Health Organization Quality of Life-BREF.
