# Supplementary figures and images for: Beneficial effect of adjuvant traditional Chinese medicine therapy on body constitution symptoms and quality of life among breast cancer patients
Source: Front Oncol. 2026 Apr 20;16:1734421. doi: 10.3389/fonc.2026.1734421 (PMC13135987; doi:10.3389/fonc.2026.1734421)

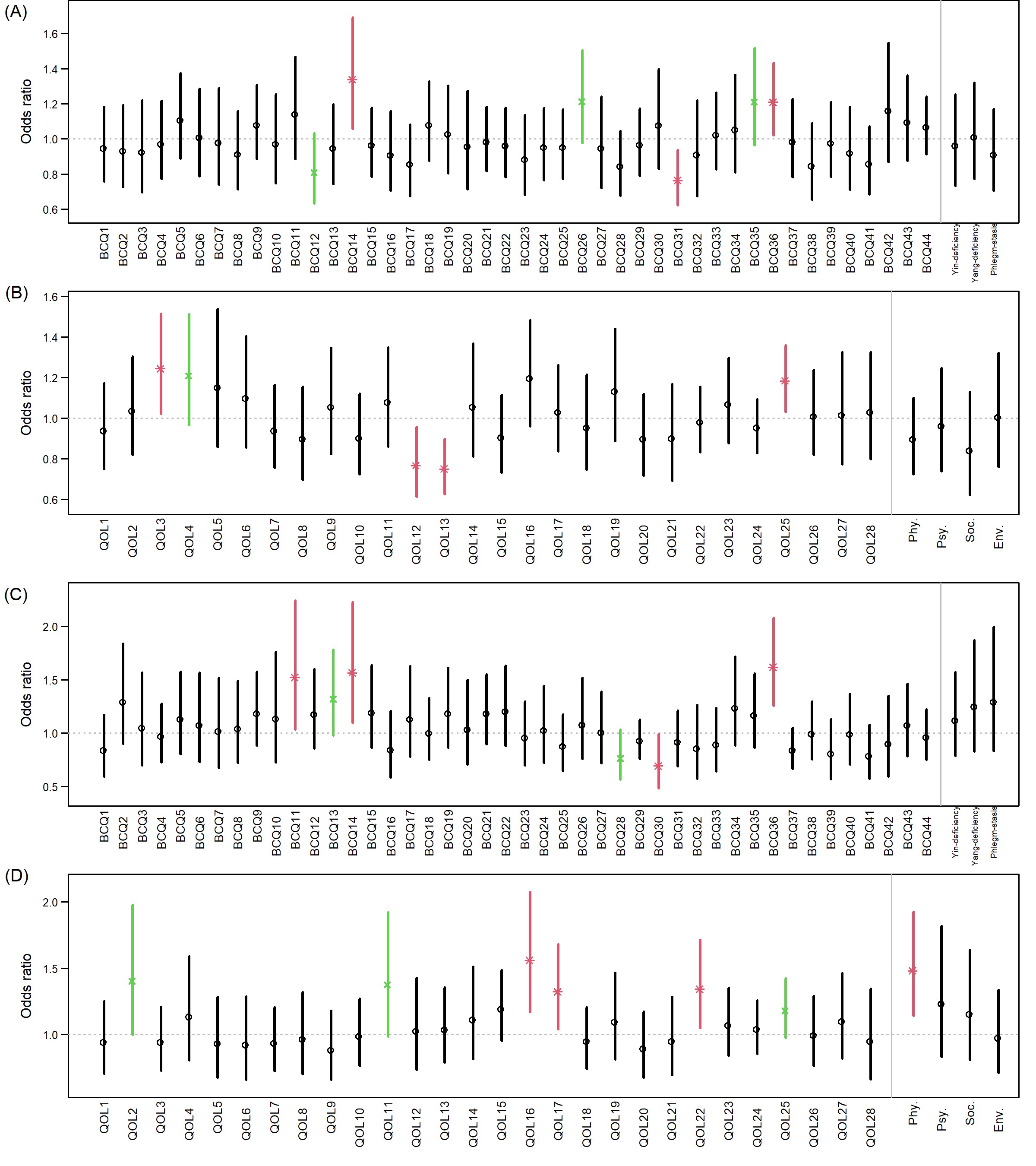

Supplement: Supplementary Figure 1 — Stage-stratified adjusted odds ratios (ORs) for improvement in BCQ and WHOQOL-BREF items associated with adjuvant CHM use. Stage-stratified analyses comparing the WM+CHM group with the WM-alone group. Panels show BCQ outcomes in (A) stage I–II and (C) stage III–IV, and WHOQOL-BREF outcomes in (B) stage I–II and (D) stage III–IV. Odds ratios (ORs) and 95% confidence intervals (CIs) were estimated using IPTW-adjusted GEE models. Exact two-sided p-values are reported; * indicates statistical significance (p < 0.05) and × indicates marginal significance (0.05 ≤ p < 0.10). Error bars represent 95% confidence intervals. BCQ, Body Constitution Questionnaire; WHOQOL-BREF, World Health Organization Quality of Life-BREF. [file Image1.jpeg]
