# Supplementary material for: Beneficial effect of adjuvant traditional Chinese medicine therapy on body constitution symptoms and quality of life among breast cancer patients
Source: Front Oncol. 2026 Apr 20;16:1734421. doi: 10.3389/fonc.2026.1734421 (PMC13135987; doi:10.3389/fonc.2026.1734421)
Supplement: Supplementary file 2 [file DataSheet1.docx]

**Supplementary Materials**

**Supplementary Table S1**. Top ten single herbs prescribed among patients receiving adjunctive Chinese herbal medicine (CHM), including Pinyin names, Chinese names, botanical nomenclature based on the *Pharmacopoeia of China (2020 edition)*, and therapeutic categories in traditional Chinese medicine.

**Supplementary Table S2**. Top ten herbal formulae prescribed among patients receiving adjunctive CHM, ranked by visit frequency. Product variants of the same classical formula were merged where appropriate.

**Supplementary Figure S1**. Stage-stratified adjusted odds ratios (ORs) for improvement in BCQ and WHOQOL-BREF outcomes associated with adjunctive CHM use. Odds ratios and 95% confidence intervals were estimated using IPTW-adjusted generalized estimating equation (GEE) models.

**Supplementary File S1**. English version of the Body Constitution Questionnaire (BCQ) used in this study.

**Supplementary File S2**. English version of the WHOQOL-BREF (Taiwan -version) questionnaire used for quality-of-life assessment in this study.
