# Supplementary material for: Beneficial effect of adjuvant traditional Chinese medicine therapy on body constitution symptoms and quality of life among breast cancer patients
Source: Front Oncol. 2026 Apr 20;16:1734421. doi: 10.3389/fonc.2026.1734421 (PMC13135987; doi:10.3389/fonc.2026.1734421)
Supplement: Supplementary file 3 [file DataSheet2.pdf]

WHOQOL-BREF (Taiwan Version)

Instructions

This assessment asks how you feel about your quality of life, health, or other areas of your life. Please answer all the questions. If you are unsure about which response to give to a question, please choose the one that appears most appropriate. This can often be your first response.

Please keep in mind your standards, hopes, pleasures and concerns. We ask that you think about your life in the **last two weeks**.

For example, thinking about the last two weeks, a question might ask:

Do you get the kind of support from others that you need?				
Not at all	Not much	Moderately	A great deal	Completely
1	2	3	4	5

You should circle the number that best fits how much support you got from others over the last two weeks. So, you would circle the number 4 if you got a great deal of support from others. Or circle number 1 if you got no support from others. Please read each question, assess your feelings, and circle the number on the scale for each question that gives the best answer for you.

1. How would you rate your quality of life?
2. How satisfied are you with your health?
3. To what extent do you feel that physical pain prevents you from doing what you need to do?
4. How much do you need any medical treatment to function in your daily life?
5. How much do you enjoy life?
6. To what extent do you feel your life to be meaningful?
7. How well are you able to concentrate?
8. How safe do you feel in your daily life?
9. How healthy is your physical environment?
10. Do you have enough energy for everyday life?
11. Are you able to accept your bodily appearance?
12. Have you enough money to meet your needs?

13. How available to you is the information that you need in your day-to-day life?
14. To what extent do you have the opportunity for leisure activities?
15. How well are you able to get around?
16. How satisfied are you with your sleep?
17. How satisfied are you with your ability to perform your daily living activities?
18. How satisfied are you with your capacity for work?
19. How satisfied are you with yourself?
20. How satisfied are you with your personal relationships?
21. How satisfied are you with your sex life?
22. How satisfied are you with the support you get from your friends?
23. How satisfied are you with the conditions of your living place?
24. How satisfied are you with your access to health services?
25. How satisfied are you with your transport?
26. How often do you have negative feelings, such as blue mood, despair, anxiety, depression?
27. Do you feel respected by others?
28. Are you usually able to get the things you like to eat?

Note: Questions 27 and 28 correspond to items in the Taiwan version of the WHOQOL-BREF

Source: Yao G, Chung CW, Yu CF, Wang JD. Development and verification of validity and reliability of the WHOQOL-BREF Taiwan version. Journal of the Formosan Medical Association. 2002;101(5):342–351.
