# Supplementary material for: Beneficial effect of adjuvant traditional Chinese medicine therapy on body constitution symptoms and quality of life among breast cancer patients
Source: Front Oncol. 2026 Apr 20;16:1734421. doi: 10.3389/fonc.2026.1734421 (PMC13135987; doi:10.3389/fonc.2026.1734421)
Supplement: Supplementary file 4 [file Table1.docx]

**Supplementary Table S1. Top ten single herbs prescribed in the present cohort**

| **Latin name (Pharmacopoeia)** | **Pinyin name** | **Chinese name** | **Botanical name** | **TCM category** | **Frequency (N)** | **Percent (%)** |
| --- | --- | --- | --- | --- | --- | --- |
| Prunellae Spica | Xia Ku Cao | 夏枯草 | *Prunella vulgaris* L. | Heat-clearing / Mass-dissipating | 1026 | 3.8647 |
| Curcumae Radix | Yu Jin | 鬱金 | *Curcuma aromatica* Salisb. | Blood-activating / Qi-regulating | 987 | 3.7178 |
| Trichosanthis Fructus | Gua Lou Shi | 栝蔞實 | *Trichosanthes kirilowii* Maxim. | Phlegm-resolving / Heat-clearing | 908 | 3.4202 |
| Taraxaci Herba | Pu Gong Ying | 蒲公英 | *Taraxacum mongolicum* Hand.-Mazz. | Heat-clearing / Detoxifying | 811 | 3.0548 |
| Astragali Radix | Huang Qi | 黃耆 | *Astragalus membranaceus* (Fisch.) Bunge | Qi-tonifying | 666 | 2.5087 |
| Polygoni Multiflori Caulis | Ye Jiao Teng | 夜交藤 | *Fallopia multiflora* (Thunb.) Harald. | Blood-nourishing / Spirit-calming | 618 | 2.3279 |
| Albiziae Cortex | He Huan Pi | 合歡皮 | *Albizia julibrissin* Durazz. | Qi-regulating / Spirit-calming | 505 | 1.9022 |
| Spatholobi Caulis | Ji Xue Teng | 雞血藤 | *Spatholobus suberectus* Dunn | Blood-activating / Blood-nourishing | 474 | 1.7855 |
| Achyranthis Bidentatae Radix | Niu Xi | 牛膝 | *Achyranthes bidentata* Blume | Blood-activating / Damp-dispelling | 434 | 1.6348 |
| Cyathulae Radix | Chuan Niu Xi | 川牛膝 | *Cyathula officinalis* K.C.Kuan | Blood-activating / Damp-dispelling | 433 | 1.6310 |

**Supplementary Table S2. Top ten herbal formulae prescribed in the present cohort**

| **Formula name (Pinyin)** | **Chinese name** | **TCM category** | **Frequency (N)** | **Percent (%)** |
| --- | --- | --- | --- | --- |
| Sheng Mai Yin | 生脈飲 | Qi- and Yin-tonifying formula | 603 | 2.2714 |
| Suan Zao Ren Tang | 酸棗仁湯 | Spirit-calming / Yin-nourishing | 254 | 0.9568 |
| Jia Wei Xiao Yao San | 加味逍遙散 | Liver-regulating / Heat-clearing | 237 | 0.8927 |
| Wen Dan Tang | 溫膽湯 | Phlegm-resolving / Gallbladder harmonizing | 166 | 0.6253 |
| Ban Xia Xie Xin Tang | 半夏瀉心湯 | Harmonizing formula | 149 | 0.5613 |
| Chai Hu Jia Long Gu Mu Li Tang | 柴胡加龍骨牡蠣湯 | Liver-regulating / Spirit-calming | 145 | 0.5462 |
| Tian Wang Bu Xin Dan | 天王補心丹 | Heart- and Yin-nourishing formula | 140 | 0.5274 |
| Shu Jing Huo Xue Tang | 疏經活血湯 | Blood-activating / Channel-unblocking | 132 | 0.4972 |
| Zhi Bai Di Huang Wan | 知柏地黃丸 | Yin-nourishing / Heat-clearing | 103 | 0.3880 |
| Gan Mai Da Zao Tang | 甘麥大棗湯 | Spirit-calming / Qi-harmonizing | 90 | 0.3390 |
